# The management of children with bronchiolitis in the Australasian hospital setting: development of a clinical practice guideline

**DOI:** 10.1186/s12874-018-0478-x

**Published:** 2018-02-12

**Authors:** Sharon O’Brien, Sally Wilson, Fenella J. Gill, Elizabeth Cotterell, Meredith L Borland, Edward Oakley, Stuart R Dalziel, on behalf of the Paediatric Research in Emergency Departments International Collaborative (PREDICT) network, Australasia

**Affiliations:** 1 Child and Adolescent Health Service, Princess Margaret Hospital for Children, Roberts Road Subiaco, Perth, WA 6008 Australia; 2 School of Nursing, Midwifery and Paramedicine, Faculty of Health Sciences, Curtin University, Perth, WA Australia; 3 School of Rural Medicine, University of New England, Armidale, NSW 2351 Australia; 4 School of Paediatric and Child Health, School of Primary, Aboriginal and Rural health Care, University of Western Australia, Perth, WA 6840 Australia; 5 Royal Children’s Hospital Melbourne, Murdoch Children’s Research Institute, 50 Flemington Rd, Parkville, VIC 3052 Australia; 6 University of Melbourne, Cnr Grattan Street & Royal Parade, Melbourne, VIC 3010 Australia; 7 Paediatric Emergency Medicine Centre of Research Excellence, Melbourne, Australia; 8 Children’s Emergency Department, Starship Children’s Hospital, Private Bag 92024, Auckland, 1142 New Zealand

**Keywords:** Bronchiolitis, Guideline, Infant, Management, Respiratory, Viral infection, Baby, Paediatric, Child, Emergency department, Hospital

## Abstract

**Background:**

Bronchiolitis is the commonest respiratory infection in children less than 12 months and cause of hospitalisation in infants under 6 months of age in Australasia. Unfortunately there is substantial variation in management, despite high levels of supporting evidence. This paper reports on the process, strengths and challenges of the hybrid approach used to develop the first Australasian management guideline relevant to the local population.

**Method:**

An adaption of the nine steps recommended by the National Health and Medical Research Council (NHMRC) and the Grading of Recommendations Assessment, Development and Evaluation (GRADE) methodology were utilised. Following establishment of the Guideline Development Committee (GDC), we identified the population, intervention, comparator, outcomes and time of interest (PICOt) questions, undertook a systematic literature search and graded the evidence and recommendations using the NHMRC and GRADE processes. Using Nominal Group Techniques (NGT), consensus was sought in formulating the clinical practice recommendations and practice points. Key health professional bodies were consulted to ensure relevance in the Australasian emergency and ward settings.

**Results:**

From 33 PICOT questions, clinical recommendations for practice that were deemed relevant to the Australasian population were identified. Specific considerations for the management of Australian and New Zealand indigenous infants in relation to the use of azithromycin and risk factors for more serious illness are included.

Using NGT, consensus demonstrated by a median Likert score > 8 for all recommendations was achieved. The guideline presents clinical guidance, followed by the key recommendations and evidence review behind each recommendation.

**Conclusion:**

Developing evidence-based clinical guidelines is a complex process with considerable challenges. Challenges included having committee members located over two countries and five time zones, large volume of literature and variation of member’s knowledge of grading of evidence and recommendations. The GRADE and NHMRC processes provided a systematic and transparent approach ensuring a final structure including bedside interface, and a descriptive summary of the evidence base and tables for each key statement. Involvement of stakeholders who will ultimately be end-users as members of the GDC provided valuable knowledge. Lessons learnt during this guideline development process provide valuable insight for those planning development of evidence-based guidelines.

**Electronic supplementary material:**

The online version of this article (10.1186/s12874-018-0478-x) contains supplementary material, which is available to authorized users.

## Background

Bronchiolitis, the commonest lower respiratory tract infection in children less than 12 months of age, is the most frequent cause of hospitalisation in infants under six months of age [1, 2] and can be a life-threatening illness of infancy. In Australia, approximately 13,500 children are admitted to hospital with bronchiolitis each year. Bronchiolitis accounted for 56% of all admissions to Australian hospitals of infants aged less than one year in 2000/01 [3]. Groups most disadvantaged in our society are disproportionately affected by bronchiolitis; in New Zealand between 2003 and 2007, the rate of bronchiolitis was four times greater in indigenous children than European children (rate ratio (RR) 4.31; 95% confidence interval (CI) 4.15–4.47) and was nearly five times greater in those living in the most deprived quintile than those in the least deprived quintile (RR 4.74; 95% CI 4.47–5.03) [4].

Bronchiolitis is caused by a viral infection, most commonly respiratory syncytial virus and is characterised by acute inflammation, oedema and necrosis of epithelial cells lining small airways, increased mucus production, and bronchospasm. Signs and symptoms are typically rhinitis, tachypnoea, wheezing, cough, crackles/inspiratory crepitations, use of accessory muscles, and/or nasal flaring [5], all contributing to respiratory distress, reduced oxygenation and difficulty feeding [6]. The management consists solely of supportive therapies such as supplemental oxygen and fluid replacement [6, 7]. Despite this, substantial variation in practice patterns occurs [8]. Results of an audit of the records of more than 3000 children who were admitted to seven Australasian hospitals with bronchiolitis, identified that ineffective interventions and diagnostic tools (e.g. inhaled salbutamol, inhaled epinephrine, oral glucocorticoids, chest x-ray, antibiotics) were used at least once in 27% to 48% of children [9]. The strategy to reduce this heterogeneity and use of ineffective practices was to develop an evidence based Australasian guideline for the management of infants presenting to, and admitted into, hospital with bronchiolitis.

Evidence based clinical practice guidelines translate findings from health research into recommendations for clinical practice [10]. Guidelines contain systematically developed statements that help practitioners and patient decision makers decide on appropriate health care for specific clinical circumstances [11]. Further, guidelines offer explicit recommendations for clinicians, influence the beliefs of healthcare providers and practitioners accustomed to outdated practices, improve the consistency of care, and provide authoritative recommendations that reassure practitioners about the appropriateness of their treatment plan. Evidence based guidelines clarify which interventions are of proven benefit and document the quality of the supporting data [12].

Although high quality international clinical practice guidelines for children with bronchiolitis exist for practitioners in the United States [13] and the United Kingdom [14], there are none for Australasian clinicians. Furthermore, the available international guidelines are not completely applicable to the Australasian population or setting as they do not take into consideration the differing environmental factors, prevalence and variance of respiratory diseases, the indigenous population risk or the health service delivery model [15].

At present many Australasian tertiary children’s hospitals and State Government departments have developed and utilise their own local guidelines for the management of bronchiolitis in their setting [16–19]. However, the methodology behind each of these guidelines is of low-quality with none utilising a systematic review of the literature, formal evaluation of the evidence or transparent consensus processes. There is heterogeneity between guidelines ensuing variation in practice, practitioner confusion, and increased medical costs due to inefficiency in patient management. For example, requirements for virology testing and acceptable oxygen saturation levels vary between existing local Australasian guidelines, both of which can directly contribute to increased length of hospital stay, unnecessary investigations, interventions and/or hospitalisations highlight the need for a single high-quality guideline.

An Australasian guideline for the management of bronchiolitis has the potential to standardise care for infants presenting with one of the most common conditions for children admitted to hospital. A guideline can enable professionals from different disciplines to come to an agreement about treatment and devise a quality framework against which practice can be measured, not only for tertiary hospital care but smaller regional, rural and remote health facilities. The development and implementation of an evidence based guideline has the potential to align practice standards across Australasia, allowing benchmarking, and individual institutions to focus on suitable implementation strategies for knowledge translation in the local context. With such standardisation there are expected health benefits as hospitalisation remains the primary determinant of health care expenditure in bronchiolitis [14]. A high-quality Australasian bronchiolitis clinical practice guideline will provide an important step in closing the gap between current clinical practice and best available evidence [10] and provide rigorously developed, valid and applicable recommendations for achieving the best possible outcomes [20].

Clinical guidelines can be developed by organisations, individuals, task forces or institutions that have a common aim to identify the current best practice based on the available research evidence in the wider context of patient and clinician experience. Guidelines are rarely based on research evidence alone, but generally also incorporate the consensus view of experts. Rigor and transparency are required in the process selected to develop consensus [21] and several approaches can be used. The three most commonly used methods being Nominal Group Technique (NGT), the Delphi survey, or a combination/hybrid approach [22].

The NGT can involve approximately eight to twelve people who identify the questions to be included in a guideline, express their views in private, and then discuss areas of disagreement. After the discussion the group members again provide their views in private to the steering committee or organisers who analyse the responses to achieve group consensus. This process allows all group members the opportunity to express their views, reduces the risk of misunderstandings whilst identifying the reasons for differences of opinion, and is a structured, transparent, and replicable way of synthesising individual judgments [23].

A Delphi method involves two or more rounds of postal or electronic questionnaires to obtain consensus or expert opinion. This method allows involvement of a larger and more geographically dispersed group of participants and avoids the risk of some individuals exercising undue influence [24]. The anonymity is a positive feature of the Delphi method in that participants do not meet face-to-face and therefore can present and react to ideas unbiased by the identity of others. However, a Delphi survey has limited opportunity for group discussion, clarification and resolution of differences of opinion. The Delphi method also has reduced reliability in comparison to the NGT [25].

Hybrid approaches occur where a combination of questionnaire and face-to-face meetings are utilised to reach consensus. This method can allow for greater inclusion of stakeholder viewpoints [26] but can still support anonymised voting on specific predetermined aspects of a clinical guideline. Further discussion can then take place to reach consensus without individuals having to declare their own preference, therefore providing a safer environment for discussion [22].

A key determinant of an evidence based guideline is the methodological approach used to assess the quality of the research reviewed. Assessment of quality directly guides the strength of the final recommendations for practice. There are several internationally accepted approaches for assessing and grading quality of evidence. Examples of these processes are the Grading of Recommendations Assessment, Development and Evaluation (GRADE), National Health and Medical Research Council (NHMRC) Evaluation of Evidence process, Strength of Recommendation Taxonomy (SORT) and the Scottish Intercollegiate Guideline Network (SIGN) [27]. Grading both the quality of the evidence and the strength of the recommendations has benefits to health care. Users of the guidelines or practice recommendations need to know how much confidence they can apply to the underlying evidence and recommendations cited within a guideline, therefore a systematic and explicit approach for review and judgment of the quality of evidence is required [28].

The GRADE method entails an assessment of the quality of a body of evidence for each individual outcome to a given clinical question, including consideration of within-study risk of bias (methodological quality), the directness of the evidence, heterogeneity, the precision of effect estimates and the risk of publication bias [29]. The GRADE method is recognised internationally as a reliable process for reviewing the quality of evidence and is a structured process for developing and presenting summary of findings for systematic reviews and guidelines. The process is transparent and includes comprehensive criteria for downgrading and upgrading the quality of the evidence ratings for the development of recommendations [29].

The NHMRC Evaluation of Evidence process is Australian and addresses the evidence to support clinical questions such as those relating to interventions, diagnosis, prognosis, aetiology and screening which are specifically related to guideline development [30]. The NHMRC Evaluation of Evidence process includes rating five key components of the ‘body of evidence’ that contribute to each recommendation. These components are: the evidence base, including number and type of studies; the level of evidence and quality of studies (risk of bias); the consistency of the study results; the potential clinical impact of the proposed recommendation; the generalisability of the body of evidence to the target population and the applicability of the body of evidence in relation to the Australian healthcare context [10]. The development of the NHMRC process was adapted from the SIGN, GRADE and SORT processes to provide a system that aligned and complemented the NHMRC evidence dimensions and documents, giving consistency and local clarity of approach [31].

The SIGN process encompasses a system that maintains the link between the strength of the available evidence and the grade of the recommendation. The levels of evidence are based on study design and methodological quality of individual studies. Utilising the SIGN process, after synthesis of the evidence base, the Guideline Development Committee must make a subjective judgement on the recommendations. This judgement is based on their clinical experience in addition to their knowledge of the evidence base [27].

Shukla et al. [32] reviewed and evaluated over 51 evidence grading systems, with the highest scoring instruments being GRADE and SIGN. The GRADE and NHMRC processes were selected for developing the Australasian Bronchiolitis Guideline. The GRADE process was selected as this is currently being used by the World Health Organisation (WHO) and increasingly, worldwide. Further, GRADE has several advantages over other systems such as explicit definitions and sequential judgements and consideration of health benefit versus harm, burden and cost. A limitation identified by Schünemann [28] was the complexity of the GRADE System and the need for a detailed manual or software to simplify the use of the system. Rationale for selecting the NHMRC process includes this being an Australian process, endorsed by the NHMRC who is Australia’s expert body for health and medical research and promotes the development and maintenance of public and individual health standards [33]. The NHMRC process is a transparent method to formulate and grade recommendations. Further, in order to gain NHMRC approval, Australian guideline developers must comply with NHMRC standards.

This paper reports on the methodology utilised to develop a specific guideline for Australasian clinical practice; assessment of evidence using the NHMRC and GRADE processes along with a hybrid consensus approach to develop clinical recommendations in a final clinical guideline for the management of bronchiolitis in the Australasian setting. The strengths and challenges encountered are presented.

## Methods

**Fig. 1:**
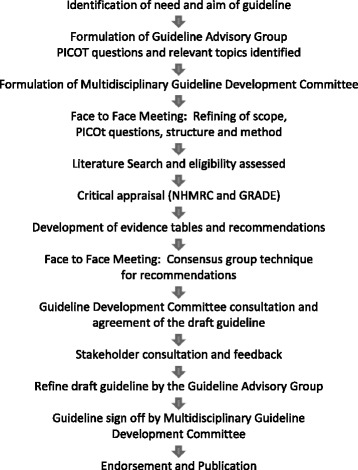
Guideline Development Process Summary

A structured guideline development process was utilised (see Fig. 1). The aim was to formulate an evidence based, clinical practice guideline for infants with bronchiolitis presenting to, and admitted into Australasian hospitals. The scope was to examine the evidence for the diagnosis and management for the purpose of improving health outcomes. The guideline addresses the emergency department (ED) and general ward management of bronchiolitis, recognising that in order to influence management for the majority of patients who present to hospital with bronchiolitis, these two areas are critical to in-hospital management. The guideline specifically excluded management in primary care and intensive care units. These exclusions were because hospitalisation is the primary determinant of health care expenditure for bronchiolitis [14] and only a small proportion of patients admitted to hospital with bronchiolitis require intensive care management [34]. It is recognised that although the guidelines exclude these two management settings the principles of management and evidence addressed in the guideline process are relevant to both settings.

The principal target group to utilise the guideline will be staff of, and health systems supporting, Australasian EDs and general paediatric wards. The guideline will be structured as a useable clinical interface with bed-side functionality, including flow diagrams and tables of key points. A descriptive summary of the evidence base and evidence based tables from the GRADE and NHMRC evidence review processes will sit behind the useable clinical interface in the final document.

A Guideline Advisory Group (GAG) was formed which consisted of the project chief investigators; three paediatric emergency physicians, one paediatrician and the project coordinator. The role of this group was to provide expert advice and contribute to the guideline development process including construction of population, intervention, comparator, outcomes and time of interest (PICOt) questions, defining the guideline scope and target audience, and oversight of the project.

Further, a multidisciplinary Guideline Development Committee (GDC) which included the members of the GAG, was convened in accordance with the NHMRC recommendations for guideline development [10]. This committee comprised of twenty-two individuals, including; eight General Paediatricians, one Paediatric Respiratory Physician, eight Paediatric Emergency Medicine Physicians, one Paediatric Intensive Care Physician, one Paediatric Nurse Practitioner, two Paediatric Nurses, and one Paediatric Emergency Nurse from a mixture of Australian and New Zealand metropolitan and non-metropolitan centres, (including representatives from six of the eight Australian States and Territories). The Australian and New Zealand Paediatric Societies were also approached seeking their representation for the GDC. This committee was established to review and synthesise the evidence to prepare the evidence based guideline, ensuring relevance to their specific speciality areas of representation.

The GAG initially identified 26 key PICOt questions and relevant topics to be included based on the American Academy of Paediatrics (AAP) 2014 bronchiolitis statement [35]. A face-to-face meeting was conducted with the GDC during which the guideline method was agreed. At this meeting current state and tertiary children’s hospitals bronchiolitis guidelines were reviewed and the original PICOt questions formulated by the GAG were refined and expanded to 33 key questions relevant to the management of bronchiolitis (Table 1).

**Table 1:** Questions answered relevant to the management of bronchiolitis

Number	Question
1	In infants presenting to hospital what factors in history and physical examination contribute to a differential diagnosis of bronchiolitis?
2	In infants presenting to hospital with bronchiolitis, what are the risk factors for admission or severe disease (e.g. prolonged length of hospital stay, intensive care unit (ICU) admission, and death)?
3	In infants presenting to hospital or hospitalised with bronchiolitis, does performing a CXR beneficially change medical management or clinically relevant end-points*?*
4	In infants presenting to hospital or hospitalised with bronchiolitis, does performing laboratory tests (blood and/or urine) beneficially change medical management or clinically relevant end-points*?*
5	In infants presenting to hospital or hospitalised with bronchiolitis, does performing virological investigations beneficially change medical management or clinically relevant end-points*?*
6	For infants presenting to hospital or hospitalised with bronchiolitis, does use of a bronchiolitis scoring system beneficially change medical management or clinically relevant end-points*?*
7	For infants presenting to hospital or hospitalised with bronchiolitis, what criteria should be used for safe discharge?
8a. i)	In infants presenting to hospital or hospitalised with bronchiolitis, does administration of Beta2 Agonists (nebulisation, aerosol, oral or IV) improve clinically relevant end-points?
8a. ii)	In older infants presenting to hospital or hospitalised with bronchiolitis, does administration of Beta2 Agonists (nebulisation, aerosol, oral or IV) improve clinically relevant end-points?
8b. i)	In infants presenting to hospital or hospitalised with bronchiolitis, with a personal or family history of atopy, does administration of Beta2 Agonists (nebulisation, aerosol, oral or IV) improve clinically relevant end-points?
8b. ii)	In older infants presenting to hospital or hospitalised with bronchiolitis, with a second or subsequent episode/s of bronchiolitis or wheeze, does administration of Beta2 Agonists (nebulisation, aerosol, oral or IV) improve clinically relevant end-points?
9	In infants presenting to hospital or hospitalised with bronchiolitis, does administration of adrenaline / epinephrine (nebulisation, IM or IV) improve clinically relevant end-points?
10	In infants presenting to hospital or hospitalised with bronchiolitis, does administration of nebulised hypertonic saline improve clinically relevant end-points?
11a.	In infants presenting to hospital or hospitalised with bronchiolitis, does administration of systemic or local glucocorticoids (nebulisation, oral, IM or IV) improve clinically relevant end-points?
11b.	In infants presenting to hospital or hospitalised with bronchiolitis, with a positive response to Beta2 Agonists, does administration of systemic or local glucocorticoids (nebulisation, oral, IM or IV) improve clinically relevant end-points?
11c.	In infants presenting to hospital or hospitalised with bronchiolitis, does administration of the combination of systemic or local glucocorticoids (nebulisation, oral, IM or IV) and adrenaline improve clinically relevant end-points?
12a.	In infants presenting to hospital or hospitalised with bronchiolitis, does administration of supplemental oxygen improve clinically relevant end-points?
12b.	In infants presenting to hospital or hospitalised with bronchiolitis, what level of oxygen saturation should lead to commencement or discontinuation of supplemental oxygen to improve clinically relevant end-points?
13.	In infants hospitalised with bronchiolitis does continuous monitoring of pulse oximetry beneficially change medical management or clinically relevant end-points?
14.	In infants hospitalised with bronchiolitis does the use of heated humidified high flow oxygen, or air, via nasal cannula improve clinically relevant end-points?
15.	In infants hospitalised with bronchiolitis, does chest physiotherapy improve clinically relevant end-points?
16a.	In infants hospitalised with bronchiolitis, does suctioning of the nose or nasopharynx improve clinically relevant end-points?
16b.	In infants hospitalised with bronchiolitis, does deep suctioning in comparison to superficial suctioning beneficially improve clinically relevant end-points?
17	In infants hospitalised with bronchiolitis, does the use of nasal saline drops improve clinically relevant end-points?
18.	In infants hospitalised with bronchiolitis, does the use of bubble CPAP improve clinically relevant end-points?
19.	In infants hospitalised with bronchiolitis, is provision of home oxygen a safe alternative for management?
20a.	In infants presenting to hospital or hospitalised with bronchiolitis, does the use of antibiotic medication improve clinically relevant end-points?
20b.	In infants presenting to hospital or hospitalised with bronchiolitis, does the use azithromycin medication improve clinically relevant end-points?
20c.	In infants presenting to hospital or hospitalised with bronchiolitis, does the use of antibiotic medication in infants who are at risk of developing bronchiectasis, improve clinically relevant end-points?
21a.	In infants presenting to hospital or hospitalised with bronchiolitis, does the use of non-oral hydration improve clinically relevant end-points?
21b.	In infants presenting to hospital or hospitalised with bronchiolitis, what forms of non-oral hydration improve clinically relevant end-points
21c.	In infants presenting to hospital or hospitalised with bronchiolitis, does limiting the volume of non-oral hydration impact on clinical relevant end-points?
22	In infants presenting to hospital or hospitalised with bronchiolitis, do infection control practises improve clinically relevant end-points?

### Search strategy

A systematic literature search for all 33 questions was undertaken. The search strategy inclusion criteria included:

- Population: infants under 24 months of age with bronchiolitis.
- Interventions: diagnostic tests and investigations, oxygen therapies, medications, rehydration, scoring systems and others that met individual research questions (Table 1)
- Comparators: no treatment as a control, standard care or treatment as usual or an alternative intervention; comparisons between different modes of delivery, frequency, dose or duration of interventions were included.
- Outcomes: length of stay, admission, readmission, death.
- Time: 1 January 2000 to 17 December 2015. Given the GAGs’ extensive experience and previous knowledge of the field, a fifteen year search range was thought to be appropriate, realising that sentinel work that had occurred prior to this would have been included in relevant Cochrane reviews and other meta-analysis.
- Language: English language.

The complete search strategy is included as Additional file 1.

Exclusion Criteria included publications of case reports, commentaries, editorials and letters, and those of infants with bronchiolitis obliterans.

The following electronic databases were searched: Ovid Medline, Ovid Embase, PubMed, Cinahl (EBSCO), Cochrane Library, Cochrane Database of Systematic Reviews (CDSR) and Cochrane Central Register of Controlled Trials (CENTRAL) for systematic reviews and randomised controlled trials (RCTs). Where systematic reviews and RCTs were not found, quasi-experimental and non-experimental observational studies (e.g. case control studies and cohort studies) were retrieved. If a systematic review published in the Cochrane Library was relevant to a question, only systematic reviews and RCTs from the year of, and years subsequent to, the documented search date in the Cochrane systematic review were included.

The study selection process was conducted in a three step process:

Step 1 – One of five members of the GAG reviewed the title and abstracts of the articles identified in the literature search. Articles that met the inclusion criteria and were relevant to any of the 33 questions were included.

Step 2 – Where screening by title and abstract was insufficient to make a decision as to relevance, a copy of the complete article was sourced and reviewed in order to make a decision regarding possible relevance. If further uncertainty existed regarding possible relevance the article was reviewed by a second person. Where there was disagreement between reviewers or uncertainty final inclusion or exclusion was decided upon by the five person GAG.

See Additional file 2 – Prisma Diagram [36].

Step 3 – Included articles were then divided into groups according to each question for which they may be relevant. Where an article was relevant to more than one question, it was placed into each appropriate group.

### Methodological assessment

Members of the GDC were divided into four working groups according to expertise to allow more effective interaction via teleconference. The 33 questions were divided into areas of clinical similarities and allocated to a single group. Each article that was potentially relevant to an individual question was independently reviewed by two members for quality and the level of evidence utilising both the GRADE [29] and NHMRC Evaluation of Evidence process [30]. Any disagreements that arose between the two reviewers were resolved through discussion with a third reviewer.

On completion of the evidence grading, recommendations were reviewed within working groups via teleconference to be included in a draft guideline. In areas with a poor evidence base, the working groups discussed the evidence base and current clinical practice and made clinical practice recommendations and practice points for a draft guideline. Alongside evidence tables, summaries of evidence were prepared for each question with review by the working groups and the GDC. Where possible, the evidence was based on systematic reviews and randomised controlled trials. Where there was only low level, unsupported evidence, clinical care statements outlining current accepted practice points were included.

Draft evidence tables and recommendations were shared electronically with all members of the GDC. A face-to-face meeting was then conducted where each recommendation was presented and all GDC members given the opportunity to present their view without interruption. Discussion ensued and any alterations were made before GDC members were requested to vote. To ascertain consensus from the multidisciplinary GDC, NGT principles [37] were used. Voting was confidential and conducted using a Likert scale (where 1–3 = disagree, 4–6 = neutral and 7–9 = agreement) for each recommendation and practice point with consensus of agreement set as a median score of 7 as reported by Rolls and Elliott [38] for each recommendation. This process led to a series of statements which were structured into a succinct document suitable for use at the patient bedside.

A draft guideline was produced including flow diagrams of patient care for infants with bronchiolitis, and highlighting key assessment and treatment points throughout the patient journey from ED presentation to ward admission, inpatient stay and discharge. The structure for the draft guideline was agreed by all members of the GDC.

The final step was to undertake stakeholder/knowledge user consultation. Formal feedback was sought from Australian and New Zealand professional medical and nursing colleges (which included: Australian Paediatric Society, New Zealand Paediatric Society, Australasian College of Emergency Medicine (ACEM), Royal Australasian College of Physicians (RACP), New Zealand Emergency Medicine Network, Australian College of Children and Young Peoples Nurses (ACCYPN), New Zealand Nursing Council and Children’s Healthcare Australasia (CHA), in addition to the clinical leads of general paediatrics and ED’s at Australian and New Zealand tertiary paediatric hospitals and consumers (Royal Children’s Hospital Family Advisory Committee). All feedback was reviewed by the GAG and changes and corrections were made if deemed appropriate. This review was the final consultation between researchers and clinicians (knowledge users) to ensure relevance of the final guideline to the Australasian emergency and paediatric ward setting.

## Results

Utilising the GRADE and NRMRC processes for assessing evidence and a hybrid NGT method to achieve consensus recommendations, an evidence based Australasian Guideline for the management of Bronchiolitis in infants was developed. From the 33 PICOT questions, clinical recommendations for practice that were deemed relevant to the Australasian population were identified and are included in the guideline. Specific considerations for the management of Australian and New Zealand indigenous infants in relation to the use of azithromycin and risk factors for more serious illness are included. Utilising the NGT method to ascertain consensus amongst the GDC (*n* = 20) resulted in achievement of a median Likert score > 8 for all recommendations. The guideline is presented in such a way that the important clinical guidance is at the front, followed by the key recommendations and the evidence review behind each of these recommendations.

## Discussion

Developing evidence based clinical guidelines is a complex process. While some aspects of the process were relatively simple, other areas provided challenges. The key issues were establishing and sustaining stakeholder engagement, management of literature, utilising the grading systems by novice users and management of communication across 5 time zones.

### Stakeholder engagement:

Early and ongoing consultation with stakeholders can be a major contributing factor to the success of a guideline development process [39]. Therefore, the GDC was established seeking diversity of specialisation and ensuring adequate opportunities for discussion and input. A wide representation of clinical experts from rural, metropolitan, secondary and tertiary paediatric hospitals, with geographical representation across Australia and New Zealand, were members of the GDC. Attracting representation and members of the committee was relatively easy, indicating that the development of an Australasian Guideline for bronchiolitis was of interest and perceived as important and timely to improve management for infants presenting to hospital with bronchiolitis. The initial face-to-face meeting enabled team introductions and specific interest areas to be identified prior to dividing the committee into smaller working groups where communication was primarily via email and teleconference. Despite challenges of geographical disparity, committee members were motivated and had effective communication skills, both of which were necessary to maintain momentum for achievement of the outputs required to meet the timeline agreed upon for the guideline development.

Given the engagement of stakeholders throughout the project, we anticipate implementation relevant to needs and widespread translation into practice [40].

### Management of literature

Despite partnering with a librarian, the volume and management of the literature was challenging. The literature search was conducted as one search encompassing all of the questions due to the expected large volume of articles which were relevant to more than one question. Notwithstanding utilising one search for all questions, over 12,000 articles were identified. Of these 3813 were duplicates, resulting in a total of 8717 articles for screening for eligibility. As a consequence, the initial review of this number of articles was time consuming but perceived as necessary to be undertaken by only the five members of the Advisory Group to ensure consistency. Getting the appropriate balance between the sensitivity of the search and precision [41] in retrieving relative articles was assisted by grouping the review questions into one search and having strict search parameters. Use of a reference management system (Endnote) was essential to organise such a large library and aided greatly in the sorting and sharing of articles between the Guideline Development group members. Lack of familiarity with using the software however, was initially problematic for some members of the Advisory and Guideline Development Groups.

### Grading systems

A further challenge was the grading of evidence for each question utilising the GRADE and NHMRC processes. This was due to many of the GDC members having minimal experience formally using these processes. Definitions for terminology relating to grading of recommendations were provided to assist with the complexity of the process. Pre-reading and education materials on the grading processes were provided and the pairings of a researcher with a clinician were deliberately undertaken to ensure shared expertise. Despite these strategies, many found the process of grading research articles unfamiliar echoing Schünemann et al. [28] experiences. Providing training on the processes may have been beneficial and could have been included at the initial face-to-face meeting of the GDC. This may have reduced the burden of explanation and teaching of these processes by the Advisory Group. Accredited GRADE training is now available locally and should be utilised.

The advantages of using the GRADE system however, outweighed the difficulties encountered. Advantages included consideration of the health benefits versus harms, burdens and costs and the development of the evidence profile and summary [28] for each question. This should assist clinicians to decide whether to use the recommendations of the guideline. In addition, the NHMRC process not only reflects the risk of bias in the study design, but takes into consideration the consistency of findings between studies, clinical impact, generalisability and applicability of the results to the local health care setting [10]. Although there is overlap of the two processes, the GRADE process provides a transparent approach which is utilised worldwide and the NHMRC process represents the latest approach recommended by the leading Australian health research body to support the translation of health and medical research into Australasian clinical practice [30]. Therefore, utilisation of both processes ensures universal understanding and acceptance internationally whilst complying with the recommendations of the NHMRC at a national level.

### Communication

Although much communication was done electronically via email, greater engagement and contribution from the group was enabled by conducting face-to-face meetings as these provided a forum for open discussion. The second face-to-face meeting not only provided a forum for discussion, but members of the GDC were given the opportunity to vote on the final clinical recommendations and achieve consensus of the recommendations. Utilising a NGT [37] to reach consensus was effective. Each recommendation contained a single focus requiring agreement or non-agreement based on the evidence collated and presented to support the recommendation. The NGT elicited a response from all members of the group present, however did not allow a voice from those unable to attend the face-to-face meeting. For the 33 questions, the range of the mean scores was 8.36–9.80 on a 10 point Likert scale, demonstrating consensus was achieved. The members unable to attend were given the opportunity following the meeting to respond via email, but they were not able to be involved actively and only aware of the discussion which occurred on the day by way of meeting minutes. The discussion that did result regarding each recommendation was largely based on suggestions for minor changes to the strength of the recommendation e.g. low to very low, rather than the statement itself.

Despite the challenges of having the GDC members located over two countries and five time zones, more than 12,000 articles to manage and review and variable knowledge base of evidence grading by reviewers, there were many positive experiences such as networking, skill and knowledge development and a sense of achievement on completion of the guideline which may contribute to the delivery of evidence based care.

## Conclusion

Development of an Australasian bronchiolitis guideline based on the NHMRC and GRADE processes was a challenging but valuable project for Australasian health providers, both medical and nursing, and health consumers. Stakeholder engagement from the onset through to completion was high, reflecting interest and the need for such a document. Involvement of stakeholders who ultimately will also be the end users, provided valuable clinical knowledge which was beneficial throughout the development process and ultimately will lead to greater likelihood of implementation of the end product [40].

Development of an evidence based national or international guideline such as this results in logistical challenges that do not occur for locally produced single institution guidelines such as geographical disparity of the committee members. A variable knowledge base of grading of evidence is a potential challenge that may be present in all situations and can have a huge impact on the process, both in relation to outcome and time. Recommendations for future guideline developers planning to use this process include: anticipating and identifying learning needs about grading processes and reference management systems; frequent regular teleconferences to maintain engagement and progress and if financially viable, additional face-to-face meetings may allow increased productivity over a shorter time period.

The final guideline structure consists of a useable clinical interface for bed-side functionality (including flow diagrams and tables of key points) with a descriptive summary of the evidence base and evidence tables (NHMRC and GRADE) for each key statement. This clinical practice guideline provides an opportunity to close the gaps between current clinical practice and the best available evidence. An implementation strategy incorporating a knowledge translation project is planned to be conducted by the PREDICT network as the next step towards consistent, evidence informed practice.

## Additional files

Additional file 1: Australasian Bronchiolitis Guideline Literature search strategy. Literature search strategy used to identify relevant articles for the guideline (DOCX 20 kb)

Additional file 2: Australasian Bronchiolitis Guideline Prisma Diagram. Prisma flow diagram of search strategy (DOCX 29 kb)

## Abbreviations

ACCYPN: Australian College of Children and Young Peoples Nurses
ACEM: Australasian College of Emergency Medicine
CHA: Children’s Healthcare Australasia
CPAP: Continuous Positive Airway Pressure
CXR: Chest x-ray
ED: Emergency Department
GAG: Guideline Advisory Group
GDC: Guideline Development Committee
GRADE: Grading of Recommendations Assessment, Development and Evaluation
HFNC: High Flow Nasal Cannulae
NGT: Nominal group technique
NHMRC: National Health and Medical Research Council
PICOt: Population, intervention, comparator, outcome and time
PREDICT: Paediatric Research in Emergency Departments International Collaborative
RACP: Royal Australasian College of Physicians
RCH: Royal Children’s Hospital Melbourne
RCT: Randomised Control Trial
SORT: Strength of Recommendation Taxonomy

## References

[1] Wohl M, Chernick V: State of the art: bronchiolitis. Am Rev Respir Dis 1978, 118:759–781. 10.1164/arrd.1978.118.4.759 212970

[2] Martinez FD (2003). Respiratory syncytial virus bronchiolitis and the pathogenesis of childhood asthma. Pediatr Infect Dis J.

[3] Roche P, Lambert S, Spencer J. Surveillance of viral pathogens in Australia: respiratory syncytial virus. Communicable Diseases Intelligence vol 27. 2003:117–22. 10.33321/cdi.2003.27.9 12725513

[4] The health status of children and young people in Auckland DHB [http://www.otago.ac.nz/nzcyes/otago085999.pdf]. Accessed 7 Feb 2018.

[5] Turner T, Wilkinson F, Harris C, Mazza D, Health for Kids Guideline Development Group (2008). Evidence based guideline for the management of bronchiolitis. Aust Fam Physician.

[6] Smyth R, Openshaw P (2006). Bronchiolitis. Lancet.

[7] Davison C, Ventre KM, Luchetti M, Randolph AG (2004). Efficacy of interventions for bronchiolitis in critically ill infants: a systematic review and meta-analysis. Pediatr Crit Care Med.

[8] Babl FE, Sheriff N, Neutze J, Borland M, Oakley E (2008). Bronchiolitis management in pediatric emergency Departments in Australia and new Zealand: a PREDICT study. Pediatr Emerg Care.

[9] Oakley E, Brys T, Borland M, Neutze J, Dalziel S (2014). Medication use in infants admitted with bronchiolitis at 7 Australian and New Zealand centres.

[10] National Health and Medical Research Council (1999). A guide to the development, implementation and evaluation of clinical practice guidelines.

[11] Field MJ, Lohr KN (1990). C**linical practice guidelines: directions for a new program**.

[12] Woolf SH, Grol R, Hutchinson A, Eccles M, Grimshaw J (1999). Potential benefits, limitations, and harms of clinical guidelines. BMJ.

[13] American Academy of Pediatrics Subcommittee on D, Management of B (2006). Diagnosis and management of bronchiolitis. Pediatrics.

[14] Ricci V, Delgado Nunes V, Murphy MS, Cunningham S (2015). Bronchiolitis in children: summary of NICE guidance. BMJ.

[15] Australian Indigenous Health Info Net: Review of respiratory disease among Indigenous peoples. In.; 2005. http://www.healthinfonet.ecu.edu.au/chronic-conditions/respiratory/reviews/our-review. Accessed 7 Feb 2018.

[16] Infants and children: acute management of bronchiolitis [http://www1.health.nsw.gov.au/pds/ArchivePDSDocuments/PD2012_004.pdf]. Accessed 7 Feb 2018.

[17] Bronchiolitis in children [https://www.sahealth.sa.gov.au/wps/wcm/connect/0a3fd50040d03f4d96fbbe40b897efc8/Bronchiolitis+in+Children_Aug2013.pdf?MOD=AJPERES&CACHEID=0a3fd50040d03f4d96fbbe40b897efc8]. Accessed 7 Feb 2018.

[18] Northern Territory Governmen tDepartment of Health. Bronchiolitis Management (Paediatric) RDH Guideline. Darwin, Northern Territory Government Department of Health; 2013.

[19] Paediatric acute care guideline: bronchiolitis [http://kidshealthwa.com/guidelines/bronchiolitis/]. Accessed 7 Feb 2018.

[20] Horvath AR (2009). Grading quality of evidence and strength of recommendations for diagnostic tests and strategies. Clin Chem.

[21] Raine R, Sanderson C, Black N (2005). Developing clinical guidelines: a challenge to current methods. BMJ.

[22] Leng GC, Pearson M, Macbeth F, Rawlins M, Littlejohns P (2004). Formulating consensus recommendations. Delivery Quality in the NHS 2004.

[23] Delbecq AL, Van De Ven AH (1971). A group process model for problem identification and program planning. J Appl Behav Sci.

[24] Bayley EW, Richmond T, Noroian EL, Allen LR (1994). A Delphi study on research priorities for trauma nursing. Am J Crit Care.

[25] Hasson F, Keeney S, McKenna H (2000). Research guidelines for the Delphi survey technique. J Adv Nurs.

[26] Davies S, Romano PS, Schmidt EM, Schultz E, Geppert JJ, McDonald KM (2011). Assessment of a novel hybrid Delphi and nominal groups technique to evaluate quality indicators. Health Serv Res.

[27] Harbour R, Miller J (2001). A new system for grading recommendations in evidence based guidelines. BMJ: British Medical Journal.

[28] Schünemann HJ, Fretheim A, Oxman AD (2006). Improving the use of research evidence in guideline development: 9. Grading evidence and recommendations. Health Research Policy and Systems.

[29] Guyatt GH, Oxman AD, Vist GE, Kunz R, Falck-Ytter Y, Alonso-Coello P, Schunemann HJ (2008). GRADE: an emerging consensus on rating quality of evidence and strength of recommendations. BMJ.

[30] NHMRC additional levels of evidence and grades for recommendations for developers of guidelines [https://www.nhmrc.gov.au/_files_nhmrc/file/guidelines/developers/nhmrc_levels_grades_evidence_120423.pdf]. Accessed 7 Feb 2018.

[31] Hillier S, Grimmer-Somers K, Merlin T (2011). FORM: an Australian method for formulating and grading recommendations in evidence-based clinical guidelines. BMC Med Res Methodol.

[32] Shukla V, Bai A, Milne S, Wells G (2008). Systematic review of evidence grading systems for grading levels of evidence. Z Evid Fortbild Qual Gesundhwesen.

[33] About the NHMRC [https://www.nhmrc.gov.au/about]. Accessed 7 Feb 2018.

[34] Oakley E, Borland M, Neutze J, Acworth J, Krieser D, Dalziel S, Davidson A, Donath S, Jachno K, South M (2013). Nasogastric hydration versus intravenous hydration for infants with bronchiolitis: a randomised trial. Lancet Respir Med.

[35] American Academy of Pediatrics Subcommittee on Diagnosis, Management of Bronchiolitis (2006). Diagnosis and management of bronchiolitis. Pediatrics.

[36] Moher D, Liberati A, Tetzlaff J, Altman DG. Preferred reporting items for systematic reviews and meta-analyses: the PRISMA statement. BMJ. 2009;339 PMC3090117 21603045

[37] James D, Warren-Forward H (2015). Research methods for formal consensus development. Nurse Res.

[38] Rolls KD, Elliott D (2008). Using consensus methods to develop clinical practice guidelines for intensive care: the intensive care collaborative project. Australian Critical Care.

[39] Shekelle PG, Woolf SH, Eccles M, Grimshaw J (1999). Developing guidelines. BMJ.

[40] Straus SE, Tetroe J, Graham I (2009). Defining knowledge translation. CMAJ.

[41] Higgins JP, Green S, JPT H, Green S (2011). Cochrane Handbook for Systematic Reviews of Interventions Version 5.1.0 [updated March 2011]. The Cochrane Collaboration.

