# Supplementary material for: The management of children with bronchiolitis in the Australasian hospital setting: development of a clinical practice guideline
Source: BMC Med Res Methodol. 2018 Feb 12;18:22. doi: 10.1186/s12874-018-0478-x (PMC5809867; doi:10.1186/s12874-018-0478-x)
Supplement: Supplementary file 1 — Australasian Bronchiolitis Guideline Literature search strategy. Literature search strategy used to identify relevant articles for the guideline (DOCX 20 kb) [file 12874_2018_478_MOESM1_ESM.docx]

# Additional file 1

# Australasian Bronchiolitis Guideline – Literature search strategy

| **Data Sources** | MEDLINE (OvidSP); EMBASE (OvidSP); PubMed; The Cochrane Library; CINAHL(EBSCO) |
| --- | --- |
| **MeSH terms**  **(MEDLINE OvidSP)** | **Population**  bronchiolitis/  bronchiolitis, viral/  respiratory syncytial viruses/  respiratory syncytial virus, human/  respiratory syncytial virus infections/  **AND**  **Intervention / Area of interest** natural history/ epidemiology/  exp "reproducibility of results"/  "severity of illness index"/  diagnosis, differential/  physical examination/  exp auscultation/  blood pressure determination/  exp palpation/  percussion/  pulse/  exp vital signs/  exp *respiratory tract infections/  risk factors/  "length of stay"/  patient admission/  patient discharge/  exp intensive care units, pediatric/  respiratory care units/  morbidity/  prevalence/  exp mortality/  exp diagnostic imaging/  radiography, thoracic/  exp hematologic tests/  urinalysis/  nasal lavage fluid/  emergency service, hospital/  asthma/ or hypersensitivity, immediate/  exp albuterol/ad, tu  epinephrine/ad,tu  bronchodilator agents/ad, tu  steroids/ad, tu  exp cholinergic antagonists/  or receptors, adrenergic, beta-2/  exp anti-inflammatory agents/  exp adrenal cortex hormones/  leukotriene antagonists/  saline solution, hypertonic/ad, tu  exp aerosols/  exp sodium chloride/  exp "nebulizers and vaporizers"/  exp oxygen inhalation therapy/  *oxygen/ad, st  exp oximetry/  home care services/  home care services, hospital-based/  exp blood gas analysis/  continuous positive airway pressure/  positive pressure respiration/  positive end respiratory pressure/  exp physical therapy modalities/  physical therapy specialty/  exp physiotherapy/  suction/  administration, intranasal/  exp fluid therapy/  rehydration solutions/  enteral feeding/  parenteral feeding/  intubation, gastrointestinal/  exp bacterial infections/  exp otitis media/  exp meningitis/  exp *antibacterial agents/tu  exp sepsis/  exp urinary tract infections/  tracheitis/  infection control/  exp primary prevention/  patient isolation/ |
| **Keywords**  **(PubMed/Cochrane Library)** | **Population**  (bronchiolit* OR wheez* OR (Respiratory Syncytial Virus*) OR rsv  **AND**  **Intervention / Area of interest**  history OR epidemiolog* OR "severity of illness" OR “disease severity” OR scoring system* OR diagnosis OR physical exam* OR auscultation OR "blood pressure" OR palpation* OR percussion OR pulse OR vital sign* OR Respiratory Tract Infection* OR risk factor* OR "length of stay" OR admission OR discharge OR “intensive care” OR ICU OR "respiratory care" OR morbidit* OR prevalence OR mortalit* OR "diagnostic imaging" OR ((chest OR thorac*) AND (x-ray* OR xray* OR “x ray” OR radiograph*)) OR  ((hematolog* OR haematolog* OR blood OR virolog* OR urine) AND (test OR tests OR exam* OR investigation*)) OR urinalys* OR “nasal lavage” OR ((nose OR nasal) AND (mucosa OR mucus)) OR emergency department* OR asthma* OR atopy OR atopic OR hypersensitiv* OR salbutamol OR albuterol OR ventolin OR levalbuterol OR adrenalin* OR epinephrin* OR beta2 adrenergic* OR beta2 agonist* OR ics OR corticosteroid* OR cortico steroid*OR cortico-steroid* OR glucocorticoid* OR gluco corticoid* OR gluco-corticoid* OR montelukast OR bronchodilat* OR steroid OR steroids OR cholinergic antagonist* OR cholinergic receptor* OR anti inflammatory agent* OR adrenal cortex hormone* OR leukotriene antagonist* OR leukotriene receptor* OR "hypertonic saline" OR (("sodium chloride" OR saline) AND (nebuliz* OR nebulis* OR vaporiz* OR vaporis* OR aerosol* OR intranasal OR "intra nasal" OR intra-nasal OR nasal)) OR oxygen OR “home oxygen” OR ((oximetry OR oximeter*) AND ("reproducibility of results" OR reliability OR validity OR function* OR technical specification* OR percutaneous measurement* OR blood gas analys*)) OR CPAP OR “continuous positive airway pressure” OR “positive pressure respiration” OR “positive end respiratory pressure” OR supplementa* oxygen OR “oxygen saturation” OR oxygen therap* OR oxygen treatment* OR physical therap* OR physiotherap* OR ((nasal* OR nose OR naso) AND (suction* OR toilet OR irrigation)) OR suction* OR saline drop* OR “nasal saline” OR “intranasal saline” OR deep suction* OR ((non oral OR oral) AND (feed* OR hydration OR fluid* OR solution* OR therap*)) OR azithromycin OR fluid therap* OR intravenous infusion* OR “non oral” OR rehydrat* OR “enteric feeding” OR “parenteral nutrition” OR “parenteral feeding” OR “enteral nutrition” OR oral* administ* OR bacterial infection* OR "otitis media" OR meningitis OR antibacterial agent* OR anti-bacterial agent* OR anti bacterial agent* OR antimicrobial agent* OR anti-microbial agent* OR anti microbial agent* OR antibiotic* OR sepsis OR septic OR urinary tract infection* OR tracheitis OR serious bacterial infection* OR “infection control” OR “primary prevention” OR isolation OR “patient care” |
| **Search dates** | The following databases were searched on the 17th December 2015 for references back to 1 January 2000: Medline (Ovid), Embase (Ovid) and The Cochrane Library. Ebsco Host Cinahl was searched back to 2000 for studies relating to chest physiotherapy and bronchiolitis only. PubMed was searched back to 2013 to capture E-pubs not available in Medline and back to 2000 to capture journals not indexed in Medline. |
| **Other information sources checked** | Reference lists of included studies were searched to identify additional relevant papers (i.e. snowballing references) |
| **Inclusion criteria** | (Publication type = clinical trial, all or clinical trial, phase i or clinical trial, phase ii or clinical trial, phase iii or clinical trial, phase iv or clinical trial or controlled clinical trial or guideline or meta analysis or practice guideline or randomized controlled trial or "review" or systematic reviews) or exp evidence-based medicine/  English language  yr="2000 -current"  age limit = ("newborn infant (birth to 1 month)" or "infant (1 to 23 months)") or infant* or newborn* or neonat* or babies or baby |
| **Exclusion criteria** | (Publication type = case reports or comment or editorial or letter) or exp bronchiolitis obliterans/ or bronchiolitis obliterans |
