# Supplementary material for: The management of children with bronchiolitis in the Australasian hospital setting: development of a clinical practice guideline
Source: BMC Med Res Methodol. 2018 Feb 12;18:22. doi: 10.1186/s12874-018-0478-x (PMC5809867; doi:10.1186/s12874-018-0478-x)
Supplement: Supplementary file 2 — Australasian Bronchiolitis Guideline Prisma Diagram. Prisma flow diagram of search strategy (DOCX 29 kb) [file 12874_2018_478_MOESM2_ESM.docx]

# Additional file 2

## **Australasian Bronchiolitis Guideline Prisma Diagram**

*PubMed was searched back to 2013 to capture E-pubs not available in Medline and back to 2000 to capture journals not indexed in Medline

**CINAHL was searched for studies relating to chest physiotherapy and bronchiolitis only

Records identified through search of electronic database:
Medline = 4127
Embase = 6875
PubMed* = 613
Cochrane Library = 907
CINAHL**= 10
**(n = 12535)**

## Screening

## Included

## Eligibility

## Identification

Additional records identified through reference lists:
**(n = 3)**

Duplicate records removed:

**(n = 3813)**

Abstracts screened:

**(n = 8722)**

Records excluded:

**(n = 7888)**

Full-text articles assessed for eligibility:

**(n = 834)**

Full-text articles excluded with reasons

**(n = 668)**

Studies included in synthesis:

**(n = 166)**
